# A novel promoter driving the expression of the quorum-sensing PqsE protein shows substantial differences in Pseudomonas aeruginosa and Pseudomonas paraeruginosa

**DOI:** 10.1099/mic.0.001788

**Published:** 2026-10-28

**Authors:** Luis Fernando Montelongo-Martínez, Sara E. Quiroz-Morales, Miguel Cocotl-Yañez, Gloria Soberón-Chávez

**Affiliations:** 1 Departamento de Microbiología y Parasitología, Facultad de Medicina, Universidad Nacional Autónoma de México, Ciudad Universitaria CP 04510, Ciudad de México, Mexico; 2 Departamento de Biología Molecular y Biotecnología, Instituto de Investigaciones Biomédicas, Universidad Nacional Autónoma de México, Tercer Circuito Exterior, Ciudad Universitaria, CP 04510, Ciudad de México, Mexico; 3 Programa de Genómica Evolutiva, Centro de Ciencias Genómicas, Universidad Nacional Autónoma de México, Cuernavaca, CP 62210, Mexico

**Keywords:** PqsE, *Pseudomonas aeruginosa* quorum-sensing, *Pseudomonas paraeruginosa*, RhlR, Vfr, virulence factor production

## Abstract

*Pseudomonas aeruginosa* is an important bacterial pathogen due to its production of different virulence factors, several of which, like rhamnolipids elastase and pyocyanin (PYO), are regulated by the quorum-sensing (QS) response, which is composed of three transcriptional regulators/autoinducer systems. At the top of the regulatory cascade is LasR/3-oxo-dodecanoyl homoserine lactone, which activates the expression of the RhlR/butanoyl homoserine lactone system and of PqsR. The latter, when coupled with its autoinducer 2-heptyl-3,4-dihydroxyquinoline (PQS), activates the transcription of the *pqsABCDE* operon, which encodes the enzymes responsible for the synthesis of 4-hydroxy-2-heptylquinoline which is the precursor of PQS. The Pqs system plays a key role in the production of PYO, mainly by regulating the expression of PqsE. The PqsE protein forms a protein complex with RhlR required to activate the transcription of the *phz* operons for PYO production. This hierarchical QS regulatory network is conserved in the recently described *Pseudomonas paraeruginosa* species, which produces the same virulence factors as *P. aeruginosa*. In this work, we characterize a promoter that is located directly upstream of *pqsE* in *P. aeruginosa* PAO1 and show that transcription from this promoter is repressed by the global regulator Vfr. By contrast, in *P. paraeruginosa* strains, whereas the −10 and −35 promoter sequences are conserved, the Vfr-binding site is not present, enabling the constitutive expression of the *pqsE* ORF independent of PqsR/PQS regulation. The implications for PYO production as a virulence-related trait in *P. paraeruginosa* are discussed.

## Graphical Abstract

A *pqsE* proximal promoter is conserved in *Pseudomonas aeruginosa* and in *Pseudomonas paraeruginosa*. It is repressed by the CRP global transcriptional regulator Vfr only in *P. aeruginosa* strains.

**Impact Statement**

*Pseudomonas aeruginosa* causes infections that are very difficult to treat due to its high antimicrobial resistance. The expression of several virulence factors is regulated by quorum sensing (QS), which includes three transcriptional factor/autoinducer systems: LasR/3-oxo-dodecanoyl homoserine lactone, RhlR/butanoyl homoserine lactone and PqsR/2-heptyl-3,4-dihydroxyquinoline (PQS). PqsR/PQS activates the expression of the *pqsABCDE* operon, and PqsE is crucial to produce pyocyanin (PYO), a key toxin. The isolation of strains carrying natural mutation in *lasR* is frequent, but in these mutants, the production of virulence factors is RhlR-dependent. Recently, the genetically divergent phylogroup 3 has been reclassified as *P. paraeruginosa*, although the members of this new species are phenotypically indistinguishable from classic *P. aeruginosa* strains. *P. paraeruginosa* possesses a conserved QS response; however, they manifest a high frequency of natural *lasR* and *pqsR* mutations, raising the question of how *P. paraeruginosa* strains can be virulent and able to produce PYO if they do not express *pqsABCDE*. Here, we show that, in *P. aeruginosa* PAO1, a promoter upstream of *pqsE* is repressed by the global regulator Vfr and that *pqsA-E* expression is largely controlled by PqsR/PQS-dependent regulation. By contrast, in *P. paraeruginosa* strains, the Vfr-binding site is absent, so *pqsE* expression is constitutive and PqsR-independent.

## Data Summary

The authors confirm that all supporting data, code and protocols have been provided within the article or through supplementary data files.

## Introduction

*Pseudomonas aeruginosa* is an opportunistic pathogen that represents an important health hazard due to its production of virulence factors and its high intrinsic and acquired antimicrobial resistance [1]. Therefore, it has been classified as a high-priority infectious agent by the World Health Organization [2]. The infections caused by *P. aeruginosa* are a primary contributor towards morbidity and mortality in people with cystic fibrosis, burn wounds and intensive care patients, among others [3, 4].

In 2010, genomic analysis of a multidrug-resistant *P. aeruginosa* strain called PA7 led to the identification of a new phylogroup that was genetically divergent from classical *P. aeruginosa* strains [5]. When the genomes of hundreds of *P. aeruginosa* isolates, including clinical and environmental strains, were analysed, it was apparent that the highly divergent PA7 clade was represented by a small number of clinical and environmental strains of diverse geographical origin. These outlier strains showed high phenotypic similarity with a minor phylogroup called phylogroup 5, although they are very distantly related [6, 7].

The genetic diversity of the strains belonging to the PA7 clade formed the basis to reclassify them as a new species called *Pseudomonas paraeruginosa* [8]. However, recently, it was reported by two independent studies, each analysing 136 *P*. *aeruginosa* clinical strains from Mexico City [9, 10], that a small number of isolates identified as belonging to the PA7 phylogroup (*P. paraeruginosa*) were indistinguishable from other classical strains based on their phenotype and by the type(s) of infection they caused.

The production of several of the *P. aeruginosa* virulence factors, including elastase, rhamnolipids and pyocyanin (PYO), is regulated at the level of transcription by the quorum-sensing (QS) response [1] that consists of a hierarchical network, which is headed by the transcriptional regulator LasR. When LasR couples with its cognate autoinducer (AI) 3-oxo-acyl-homoserine lactone (3O-C_12_-HSL, produced by LasI), it activates the transcription of some virulence factors, including elastase. LasR also activates expression of RhlR, a second LuxR-family transcriptional regulator. When bound to its cognate AI, butanoyl-homoserine lactone (C_4_-HSL, produced by RhlI) RhlR activates the transcription of genes encoding enzymes involved in rhamnolipid biosynthesis. RhlR is also required for expression of the duplicated operons *phzA1-G1* and *phzA2-G2*, which encode the enzymes of PYO synthesis.

A third QS system, called Pqs [11], consists of the transcriptional regulator PqsR, whose expression is also activated by LasR/3O-C_12_-HSL. When bound to its cognate AI, 2-heptyl-3,4-dihydroxyquinoline (PQS), PqsR binds to the promoter in front of *pqsA* and activates transcription of the *pqsABCDE* operon. This operon encodes enzymes responsible for the synthesis of a precursor of PQS, 4-hydroxy-2-heptylquinoline (HHQ). One of the *pqsABCDE* ORFs, PqsE, has thioesterase activity (required in some strains for HHQ synthesis [12]) and also forms a complex with RhlR [13]. This complex is required for activation of the *phzA-G* operons, and hence for PYO production [14].

It has been reported that *P. paraeruginosa* strains ATCC 9027 [15] and MAZ105 [16] contain mutations in the Pqs QS system and are thus unable to express the entire *pqsABCDE* operon. However, PqsE is expressed in these strains (and PYO is accordingly produced) under certain culture conditions, indicating that expression of *pqsE* can, in some circumstances, be independent of PqsR binding to the *pqsA* promoter. The promoter driving *pqsE* expression in these strains has not been identified, though. PqsR-independent expression of *pqsE* seems to be very common among *P. paraeruginosa* strains (including the PA7 strain [5]), as judged by the high frequency of natural mutations that affect the Pqs QS system [16].

Vfr is a *P. aeruginosa* global regulator of the cAMP receptor protein (CRP) family [17] which regulates the expression of several regulatory genes involved in the expression of virulence-associated traits, including LasR [18] and RhlR [19]. In *P. paraeruginosa*, Vfr and cAMP are also involved in the expression of the *exlBA* operon encoding the cytotoxic exolysin A [20].

In this work, we identify a *pqsE* promoter located at 213 nucleotides upstream from the ATG translational start site in both *P. aeruginosa* PAO1 and *P. paraeruginosa* MAZ105, which is independent of PqsR. We further show that in PAO1, this promoter contains a Vfr-binding site that leads to repression of *pqsE* expression, whereas in MAZ105, the Vfr-binding site is absent, allowing *pqsE* expression independent of Vfr. The nucleotide sequence of this *pqsE* promoter region reported here is highly conserved among *P. aeruginosa* and *P. paraeruginosa* strains suggesting a key role in the regulation of this protein and hence in PYO production.

## Results and discussion

### Identification of a proximal *pqsE* promoter in *P. aeruginosa* PAO1 and *P. paraeruginosa* MAZ105

*In silico* analysis of the nucleotide sequence upstream of the *pqsE* ORF in the PAO1 strain using the BPROM software (Softberry) revealed a putative sigma 70 promoter with −10 and −35 RNA polymerase-binding elements located at −213 nucleotides from *pqsE* translational start site (considering that the −213 nucleotide is the first nucleotide of the predicted -10 box), as well as a putative Vfr-binding site (Fig. 1). Notably, the predicted Vfr-binding site overlaps with the −35 motif in the promoter sequence, suggesting that transcription from this promoter is repressed by the CRP homologue (Fig. 1). Analysis of the same region in the environmental strain MAZ105 showed that the sequence and position of the −10 and −35 RNA polymerase-binding elements were conserved. However, the predicted Vfr-binding site in MAZ105 exhibited lower similarity with the reported Vfr-box core sequence [21] compared with the corresponding region in PAO1 (Fig. 1). The putative promoter and Vfr-binding sequences are both located within the *pqsD* coding sequence, although the nucleotide differences in the putative Vfr-binding motif in *P. aeruginosa* PAO1 and its absence in *P. paraeruginosa* MAZ105 do not affect the PqsD amino acid sequence. It is noteworthy that the nucleotide sequence of the *pqsE* upstream region is extremely conserved between *P. aeruginosa* PAO1 and *P. paraeruginosa* MAZ105 (93% identity) and that one region where the nucleotide changes are clustered corresponds to the Vfr-binding site in strain PAO1 (Fig. 1).

**Fig. 1.**
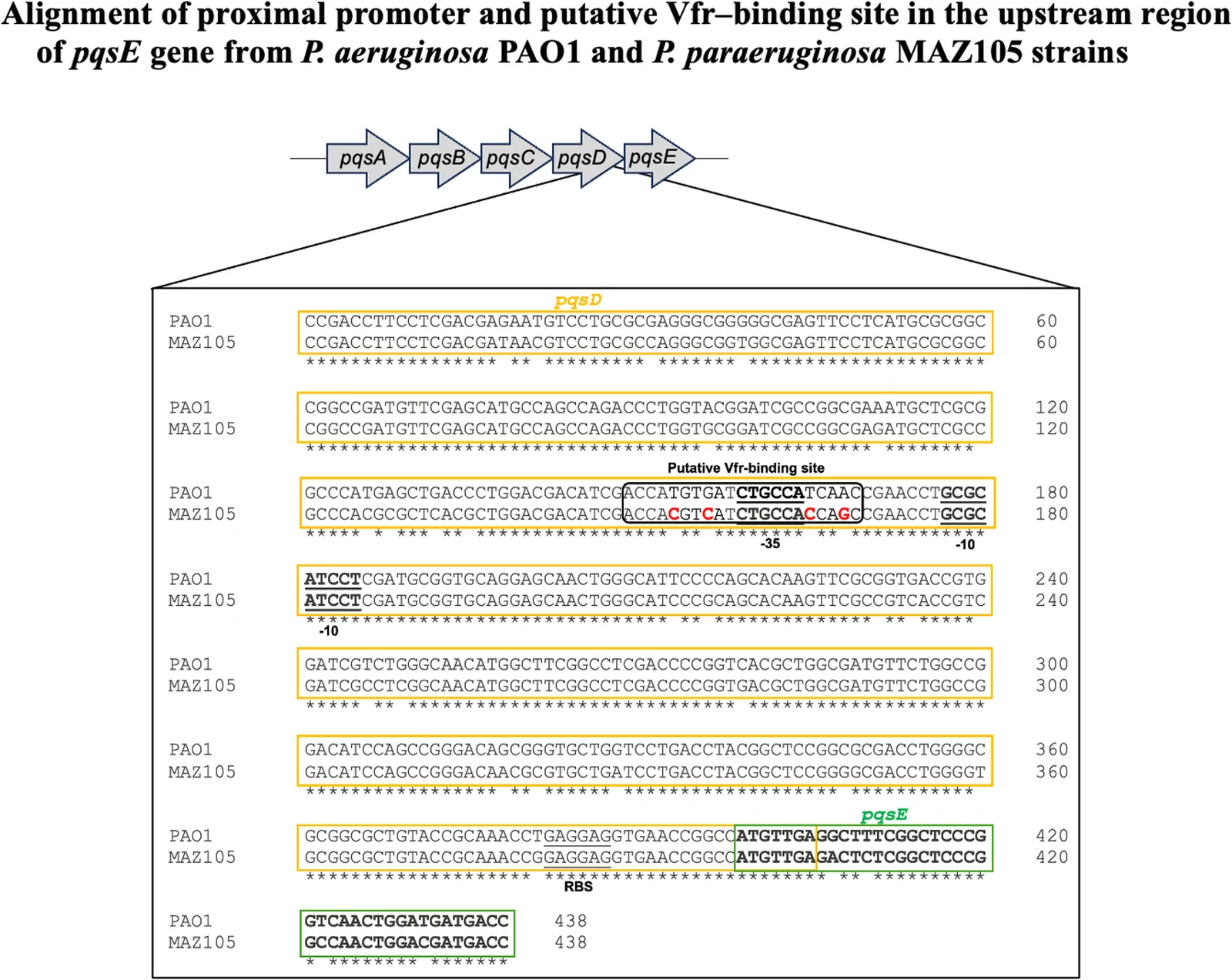
Identification of a proximal *pqsE* promoter in *P. aeruginosa* PAO1 and *P. paraeruginosa* MAZ105. Nucleotide sequence alignment of the *P. aeruginosa* PAO1 and *P. paraeruginosa* MAZ105 *pqsE* upstream region. The predicted RNA-polymerase −10 and −35 sequences are indicated, as well as a putative Vfr-binding site that overlaps the −35 region. This alignment shows that the MAZ105 strain lacks four nucleotides previously reported to be important for Vfr binding (shown in red).

### The identified *pqsE* promoter is functional and is repressed by Vfr in *P. aeruginosa* PAO1 but not in *P. paraeruginosa* MAZ105

To determine the functionality of the identified promoter proximal to *pqsE*, *lacZ* transcriptional fusions were constructed by cloning the promoter regions from PAO1 and MAZ105 strains into pLP170 [22], generating plasmids pLPpqsE^PAO1^ and pLPpqsE^MAZ105^, respectively (Fig. 2a). These plasmids were introduced into PAO1 and into an otherwise isogenic PAO1-derived *vfr* deletion mutant to determine transcriptional activity from the promoter regions in the two different genetic backgrounds. Transcriptional activity was measured by monitoring *β*-galactosidase levels, as previously described [23]. The PAOΔ*vfr* strain (Table S1, available in the online Supplementary Material) was constructed by the double recombination method described previously [24]. All molecular biology techniques were carried out as described previously [25], and transformation of *P. aeruginosa* PAO1 and its Δ*vfr* mutant was done as reported [26]. The oligonucleotides used to generate this mutant, and the transcriptional fusions are listed in supplementary material (Table S1).

**Fig. 2.**
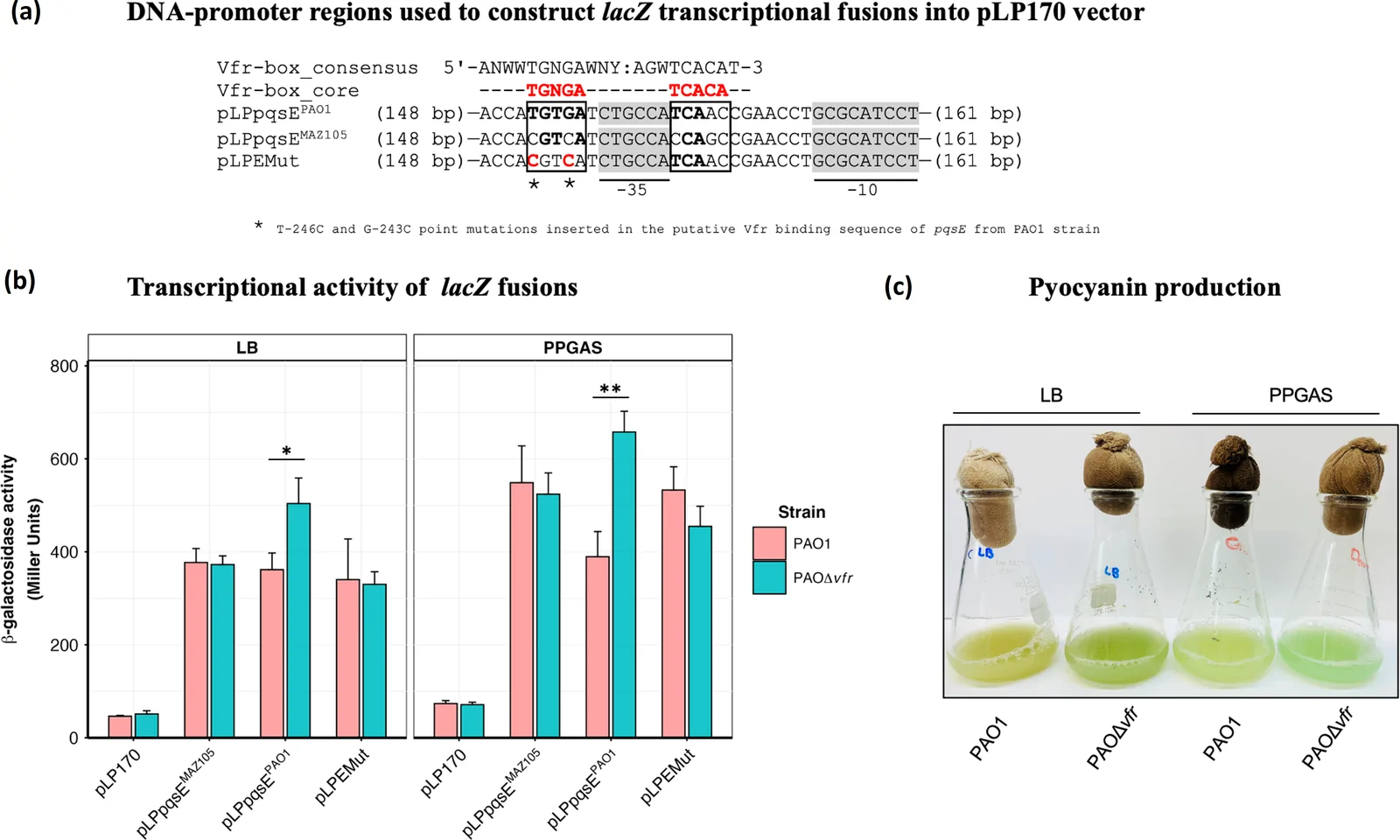
The expression of the proximal *pqsE* promoter from *P. aeruginosa* PAO1 and *P. paraeruginosa* MAZ105 differs in their repression by Vfr. (**a**) Nucleotide sequence of the proximal *pqsE* promoter regions used to construct the *lacZ* transcriptional fusions in plasmid pLP170. The sequences correspond to *P. aeruginosa* PAO1 (pLPpqsE^PAO1^), *P. paraeruginosa* MAZ105 (pLPpqsE^MAZ105^) and the pLPpqsE^PAO1^ derivative plasmid carrying point mutations T-246C and G-243C relative to the ATG translational start site of *pqsE* (pLPEMut), which disrupts the putative Vfr-binding site. (**b**) Transcriptional activity of *lacZ* fusions encoded in the pLPpqsE^PAO1^, pLPpqsE^MAZ105^ and pLPEMut plasmids, when expressed in PAO1 and PAOΔ*vfr* strains cultured in LB or PPGAS media. Bar graphs represent the mean±sd of three independent biological replicates, each comprising three technical replicates per strain. The statistically significant differences between groups were determined using an unpaired t-test (*P<0.05, **P<0.01 and ***P<0.001). (**c**) A representative image showing PYO production by PAO1 and PAOΔ*vfr* strains cultured in LB or PPGAS media.

Our data show that the expression of the PAO1-derived *pqsE::lacZ* fusion was repressed in LB medium in the presence of Vfr, whereas the fusion derived from MAZ105 was not (Fig. 2b). To further confirm that Vfr-dependent repression resulted from the binding of this transcriptional regulator to the predicted Vfr-binding site in pLPpqsE^PAO1^, we introduced a pair of mutations (T246C and G243C) that disrupt the binding site using the QuickChange site-directed mutagenesis as previously described [27], and this yielded plasmid pLPEMut (Fig. 2a). The T246C and G243C point mutations abolished Vfr-mediated repression of the PAO1-derived *pqsE* promoter. We conclude that these mutations impair recognition of the DNA motif by Vfr, resulting in transcriptional activity similar to that observed with pLPpqsE^MAZ105^ fusion. However, this derepression was not complete, as *β*-galactosidase levels did not reach those observed for pLPpqsE^PAO1^ in the PAOΔ*vfr* mutant strain (Fig. 2b). Also, the production of PYO in the PAOΔ*vfr* mutant strain was increased compared to that of the PAO1 wild-type strain, presumably due to the increased expression of *pqsE* in this mutant (Fig. 2c).

It has been reported that under phosphate limiting conditions, LasR is dispensable, and the expression of QS-regulated virulence traits is dependent only on RhlR [28]. Moreover, it has been reported that *P. paraeruginosa* strains ATCC 9027 and MAZ105 are only able to produce PYO when cultured in phosphate-limited conditions, such as the PPGAS or FDA media [16, 29]. Presumably, this is because they have natural mutations in *lasR* which make them unable to express *rhlR* when cultured in LB medium [15, 16, 30]. To determine whether the level of expression of the proximal *pqsE* promoter was affected by culture medium composition, we measured *β*-galactosidase production from the PAO1- and MAZ105-derived *lacZ* fusions. This revealed that the *lacZ* fusion derived from MAZ105 showed higher expression on PPGAS medium in both the PAO1 and the PAOΔ*vfr* backgrounds, whereas expression from the PAO1-derived fusion was the same in both media (Fig. 2b). These results suggest that, in MAZ105, another factor likely contributes towards regulating transcription from the proximal *pqsE* promoter and that this factor responds to medium composition. We could not identify any putative binding site(s) for the two-component transcriptional regulator PhoB (PhoB is known to regulate expression from the *rhlR* promoter region [31]) which might affect expression from the *pqsE* promoter.

To further confirm that the increased expression of the *pqsE::lacZ* encoded in pLPpqsE^PAO1^ by PAOΔ*vfr* mutant was caused by *vfr* deletion, we showed that the introduction of the PAO1 *vfr* gene, carried in plasmid pBBR1MCS-vfr (Table S1), caused its repression to the same levels as in the wild-type PAO1 strain (Fig. 3). Furthermore, as shown (Fig. 2), PAOΔ*vfr* mutant overproduces PYO compared with its progenitor, and we show that the reduced level of its production was restored by the introduction of plasmid pBBR1MCS-vfr (Fig. 3). The derepression of PAO1 *pqsE* (pLPpqsE^PAO1^) and PYO production in PAOΔ*vfr* and its complementation by pBBR1MCS-vfr was observed in PPGAS medium (Fig. 3). We were unable to determine the complementation of *pqsE::lacZ* fusion and PYO production in LB medium because the overexpression of Vfr in PAO Δ*vfr* carrying plasmids pBBR1MCS-vfr and pLPpqsE^PAO1^ resulted in variable PYO production and delayed growth. The underlying cause of this phenomenon remains unclear.

**Fig. 3.**
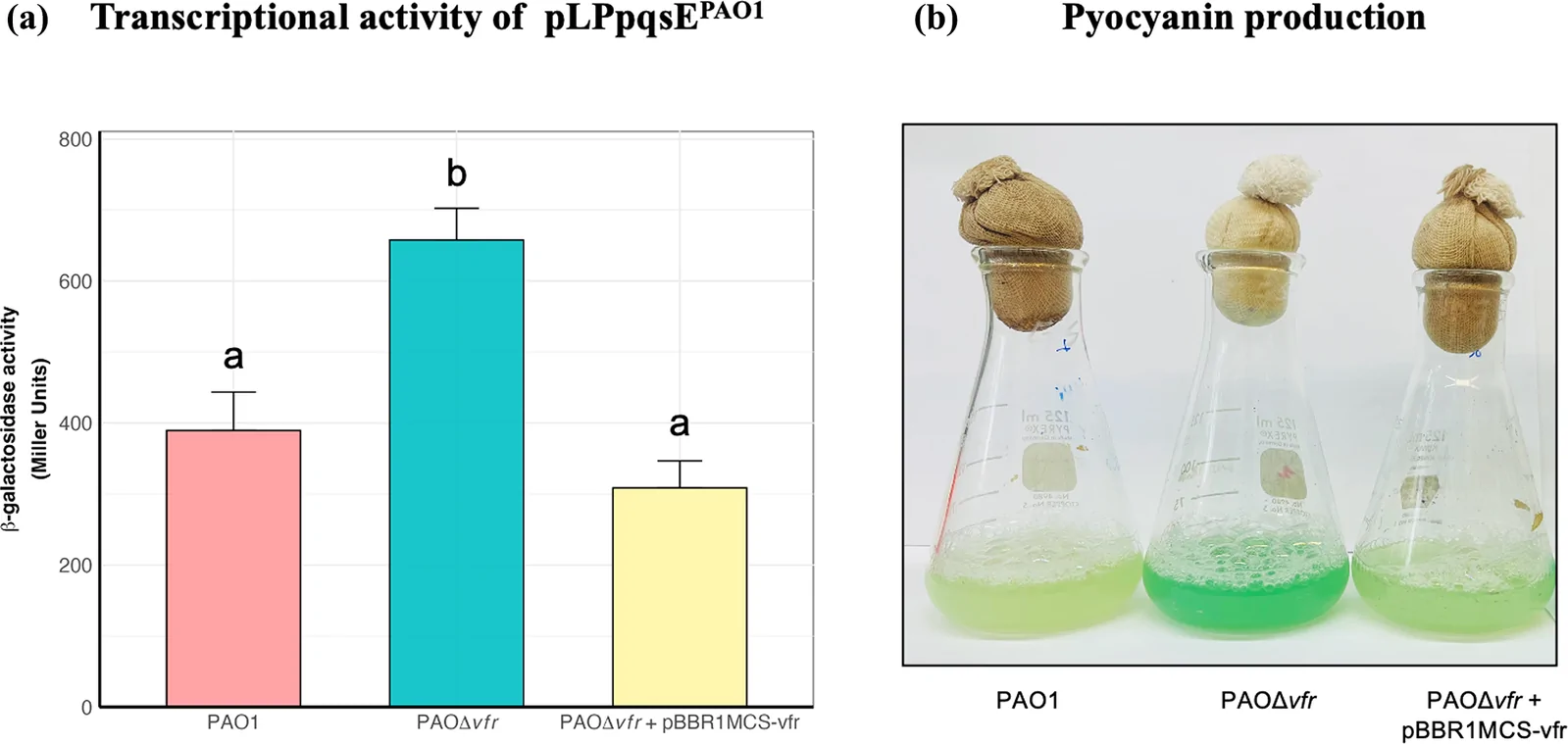
The expression of *vfr* from plasmid pBBR1MCS-vfr complements the repression of the *pqsE* proximal promoter and the hyperproduction of PYO. (**a**) Transcriptional activity of the *lacZ* fusion encoded in the pLPpqsE^PAO1^ plasmid when expressed in PAO1 and in PAOΔ*vfr* containing or not containing plasmid pBBR1MCS-vfr cultured on PPGAS medium. Bar graphs represent the mean±sd of three independent biological replicates, each comprising three technical replicates per strain. Statistically significant differences between groups were determined using one-way ANOVA followed by the Tukey HSD test (*P*<0.05). Different letters above the bars indicate statistically distinct groups, while groups sharing the same letter are not significantly different. (**b**) A representative image showing PYO production by PAO1, PAOΔ*vfr* and PAOΔ*vfr*+pBBR1MCS-vfr, all strains carrying plasmid pLPpqsE^PAO1^.

### The proximal *pqsE* promoter is conserved among different *P. aeruginosa* phylogroups and among *P. paraeruginosa* strains, but the Vfr-binding site is absent in *P. paraeruginosa* strains

The *pqsD* nucleotide sequence is highly conserved among *P. aeruginosa* strains belonging to phylogroups 1 and 2, which include more than 90% of all isolates [32]. Consequently, the proximal *pqsE* promoter and Vfr-binding site are also highly conserved.

The data reported here are consistent with our previous findings demonstrating PqsR-independent *pqsE* expression in *P. paraeruginosa* strains ATCC 9027 and MAZ105 [15, 16]. To determine whether our observation with two *P. paraeruginosa* strains represented a general phenomenon in *P. paraeruginosa*, the nucleotide sequences corresponding to the Vfr-binding site in the upstream region of *pqsE* from PAO1 and MAZ105 were used to search for homologous sequences in strains belonging to this newly defined species in the *Pseudomonas* genome database (https://www.pseudomonas.com/); accessed in March 2026) [33]. Global nucleotide alignments were performed using Clustal Omega at the EMBL-EBI server to identify mutations [34].

In addition, we search for these DNA sequences in *P. aeruginosa* phylogroup 5 strains to determine whether the Vfr-independent regulation of the proximal *pqsE* promoter was conserved among strains belonging to this phylogroup, since it has been proposed [31] that they present convergent genomic modifications with *P. paraeruginosa* that contribute to compensate their lower virulence [16, 32] caused by the deletion of genes encoding the type three secretion system and effectors.

The nucleotide alignments showed that the 48 *P*. *paraeruginosa* strains present in the database contain the same sequence as MAZ105, lacking the Vfr-binding site (Fig. S1). In contrast, this regulatory feature is conserved among *P. aeruginosa* phylogroup 5 strains, which contain the Vfr-binding site identified in the PAO1 strain (Fig. S2). These results show that the lack of a Vfr-binding site, and therefore the lack of repression of the proximal *pqsE* promoter, is unique to this newly described species, *P. paraeruginosa*. In contrast, the regulation is conserved in the *P. aeruginosa* phylogroup 5, suggesting that the regulation of the proximal *pqsE* promoter in these strains is similar to that described for PAO1, despite sharing similar physio-pathogenic characteristics with *P. paraeruginosa*.

### Concluding remarks

The proximal *pqsE* promoter described here represents a new regulatory element controlling the expression of PqsE, a central regulator of the QS response in *P. aeruginosa* and *P. paraeruginosa*. This moonlighting protein forms a complex with RhlR. This, in turn, modulates the transcriptional activity of RhlR, enabling the bacteria to produce PYO [14]. Although PqsE homologues are found in *Burkholderia*, *P. aeruginosa* and *P. paraeruginosa*, it is only in the latter two species that this protein interacts with a transcriptional factor to regulate gene expression [35]. Therefore, the existence of this newly discovered promoter potentially expands the conditions where PqsE and RhlR targets could be expressed independently of PqsR regulation.

Furthermore, our finding that the proximal *pqsE* promoter is repressed by Vfr in *P. aeruginosa*, but not in *P. paraeruginosa*, highlights a regulatory difference in virulence factor production between these bacteria. This distinction is notable because the two species are largely indistinguishable in the infections they cause and likely share a broadly similar regulatory architecture.

## Supplementary material

10.1099/mic.0.001788 Supplementary Material 1.

## Abbreviations

AI: autoinducer
C_4_-HSL: butanoyl-homoserine lactone
CRP: cAMP receptor protein
HHQ: 4-hydroxy-2-heptylquinoline
3O-C_12_-HSL: 3-oxo-acyl-homoserine lactone
PQS: 2-heptyl-3,4-dihydroxyquinoline
PYO: pyocyanin
QS: quorum sensing
