# Supplementary material for: A novel promoter driving the expression of the quorum-sensing PqsE protein shows substantial differences in Pseudomonas aeruginosa and Pseudomonas paraeruginosa
Source: Microbiology (Reading). 2026 Oct 28;172(10):001788. doi: 10.1099/mic.0.001788 (PMC13716326; doi:10.1099/mic.0.001788)
Supplement: Supplementary file 1 [file mic-172-01788-s001.pdf]

Supplementary information
A novel promoter driving the expression of the quorum-sensing PqsE protein shows substantial differences in *Pseudomonas aeruginosa* and *Pseudomonas paraeruginosa*

Authors

Luis Fernando Montelongo-Martínez^[1], Sara E. Quiroz-Morales^[2], Miguel Cocotl-Yañez[1], Gloria Soberón-Chávez[2,3]*

^LFMM and SEQM made equal contributions to this article

LFMM ORCID: 0000-0002-5807-3689

SEQM ORCID: 0009-0007-8547-3630

MCY ORCID: 0000-0002-4932-2284

GSC ORCID: 0000-0002-0282-3882

Affiliations

[1] Facultad de Medicina, Departamento de Microbiología y Parasitología, Universidad Nacional Autónoma de México, Ciudad Universitaria CP 04510, Ciudad de México, México.

[2]Departamento de Biología Molecular y Biotecnología, Instituto de Investigaciones Biomédicas, Universidad Nacional Autónoma de México, Tercer Circuito Exterior, Ciudad Universitaria, CP 04510, Ciudad de México, México.

[3]Programa de Genómica Evolutiva. Centro de Ciencias Genómicas, Universidad Nacional Autónoma de México, Cuernavaca, CP 62210, México.

Corresponding author and email address

Gloria Soberón-Chávez gloria@biomedicas.unam.mx, gsoberon@ccg.unam.mx

Figure S1. Multiple sequence alignment of the proximal *pqsE* promoter sequences in *P. paraeruginosa* strains

```
                           -35                -10
Vfr-box_core      ----TGNGA-------TCACA----------------
PAO1              ACCATGTGATCTGCCATCAACCGAACCTGCGCATCCT   37
MAZ105            ACCACGTCATCTGCCACCAGCCGAACCTGCGCATCCT   37
PA7               ACCACGTCATCTGCCACCAGCCGAACCTGCGCATCCT   37
CLJ1              ACCACGTCATCTGCCACCAGCCGAACCTGCGCATCCT   37
ATCC9027          ACCACGTCATCTGCCACCAGCCGAACCTGCGCATCCT   37
CLJ3              ACCACGTCATCTGCCACCAGCCGAACCTGCGCATCCT   37
pae832            ACCACGTCATCTGCCACCAGCCGAACCTGCGCATCCT   37
pae802            ACCACGTCATCTGCCACCAGCCGAACCTGCGCATCCT   37
pae815            ACCACGTCATCTGCCACCAGCCGAACCTGCGCATCCT   37
KCJ3K63           ACCACGTCATCTGCCACCAGCCGAACCTGCGCATCCT   37
pae413            ACCACGTCATCTGCCACCAGCCGAACCTGCGCATCCT   37
Pa5196            ACCACGTCATCTGCCACCAGCCGAACCTGCGCATCCT   37
EML1794           ACCACGTCATCTGCCACCAGCCGAACCTGCGCATCCT   37
KCJ3K52           ACCACGTCATCTGCCACCAGCCGAACCTGCGCATCCT   37
Zw26              ACCACGTCATCTGCCACCAGCCGAACCTGCGCATCCT   37
EML1796           ACCACGTCATCTGCCACCAGCCGAACCTGCGCATCCT   37
EML1795           ACCACGTCATCTGCCACCAGCCGAACCTGCGCATCCT   37
EML1793           ACCACGTCATCTGCCACCAGCCGAACCTGCGCATCCT   37
MIN-137           ACCACGTCATCTGCCACCAGCCGAACCTGCGCATCCT   37
A39-1             ACCACGTCATCTGCCACCAGCCGAACCTGCGCATCCT   37
PABL043           ACCACGTCATCTGCCACCAGCCGAACCTGCGCATCCT   37
AZPAE15042        ACCACGTCATCTGCCACCAGCCGAACCTGCGCATCCT   37
NCTC13628         ACCACGTCATCTGCCACCAGCCGAACCTGCGCATCCT   37
paerg007          ACCACGTCATCTGCCACCAGCCGAACCTGCGCATCCT   37
MRSN3705          ACCACGTCATCTGCCACCAGCCGAACCTGCGCATCCT   37
MRSN8141          ACCACGTCATCTGCCACCAGCCGAACCTGCGCATCCT   37
MRSN6241          ACCACGTCATCTGCCACCAGCCGAACCTGCGCATCCT   37
AUS217            ACCACGTCATCTGCCACCAGCCGAACCTGCGCATCCT   37
AUS483            ACCACGTCATCTGCCACCAGCCGAACCTGCGCATCCT   37
LMG5031           ACCACGTCATCTGCCACCAGCCGAACCTGCGCATCCT   37
AR_0356           ACCACGTCATCTGCCACCAGCCGAACCTGCGCATCCT   37
AR441             ACCACGTCATCTGCCACCAGCCGAACCTGCGCATCCT   37
CR1               ACCACGTCATCTGCCACCAGCCGAACCTGCGCATCCT   37
515477            ACCACGTCATCTGCCACCAGCCGAACCTGCGCATCCT   37
WH-SGI-V-07287    ACCACGTCATCTGCCACCAGCCGAACCTGCGCATCCT   37
WH-SGI-V-07261    ACCACGTCATCTGCCACCAGCCGAACCTGCGCATCCT   37
WH-SGI-V-07618    ACCACGTCATCTGCCACCAGCCGAACCTGCGCATCCT   37
WH-SGI-V-07064    ACCACGTCATCTGCCACCAGCCGAACCTGCGCATCCT   37
WH-SGI-V-07055    ACCACGTCATCTGCCACCAGCCGAACCTGCGCATCCT   37
WH-SGI-V-07370    ACCACGTCATCTGCCACCAGCCGAACCTGCGCATCCT   37
WH-SGI-V-07234    ACCACGTCATCTGCCACCAGCCGAACCTGCGCATCCT   37
WH-SGI-V-07165    ACCACGTCATCTGCCACCAGCCGAACCTGCGCATCCT   37
WH-SGI-V-07072    ACCACGTCATCTGCCACCAGCCGAACCTGCGCATCCT   37
ATCC33359         ACCACGTCATCTGCCACCAGCCGAACCTGCGCATCCT   37
EML548            ACCACGTCATCTGCCACCAGCCGAACCTGCGCATCCT   37
EML528            ACCACGTCATCTGCCACCAGCCGAACCTGCGCATCCT   37
EML545            ACCACGTCATCTGCCACCAGCCGAACCTGCGCATCCT   37
AZPAE14901        ACCACGTCATCTGCCACCAGCCGAACCTGCGCATCCT   37
AZPAE14941        ACCACGTCATCTGCCACCAGCCGAACCTGCGCATCCT   37
                  **** ** ******** ** *****************
```

Figure S2. Multiple sequence alignment of the Vfr-binding site and the proximal *pqsE* promoter sequences in *P. aeruginosa* phylogroup 5 strains

```
                                     -35                 -10
Vfr-box_core          ----TGNGA-------TCACA----------------
PAO1                  ACCATGTGATCTGCCATCAACCGAACCTGCGCATCCT   37
MAZ105                ACCACGTCATCTGCCACCAGCCGAACCTGCGCATCCT   37
PA-W1                 ACCATGTGATCTGCCATCAACCGAACCTGCGCATCCT   37
185345                ACCATGTGATCTGCCATCAACCGAACCTGCGCATCCT   37
014-2A                ACCATGTGATCTGCCATCAACCGAACCTGCGCATCCT   37
VET-121               ACCATGTGATCTGCCATCAACCGAACCTGCGCATCCT   37
ENV-681               ACCATGTGATCTGCCATCAACCGAACCTGCGCATCCT   37
IIVV-SD1              ACCATGTGATCTGCCATCAACCGAACCTGCGCATCCT   37
PALA52                ACCATGTGATCTGCCATCAACCGAACCTGCGCATCCT   37
AV15                  ACCATGTGATCTGCCATCAACCGAACCTGCGCATCCT   37
PA0002                ACCATGTGATCTGCCATCAACCGAACCTGCGCATCCT   37
ENV-552               ACCATGTGATCTGCCATCAACCGAACCTGCGCATCCT   37
H09                   ACCATGTGATCTGCCATCAACCGAACCTGCGCATCCT   37
105/4                 ACCATGTGATCTGCCATCAACCGAACCTGCGCATCCT   37
ROUE01                ACCATGTGATCTGCCATCAACCGAACCTGCGCATCCT   37
8                     ACCATGTGATCTGCCATCAACCGAACCTGCGCATCCT   37
AUS226                ACCATGTGATCTGCCATCAACCGAACCTGCGCATCCT   37
Zw73_1                ACCATGTGATCTGCCATCAACCGAACCTGCGCATCCT   37
856                   ACCATGTGATCTGCCATCAACCGAACCTGCGCATCCT   37
VET-58                ACCATGTGATCTGCCATCAACCGAACCTGCGCATCCT   37
ENV-567               ACCATGTGATCTGCCATCAACCGAACCTGCGCATCCT   37
VET-60                ACCATGTGATCTGCCATCAACCGAACCTGCGCATCCT   37
VET-73                ACCATGTGATCTGCCATCAACCGAACCTGCGCATCCT   37
ENV-566               ACCATGTGATCTGCCATCAACCGAACCTGCGCATCCT   37
ENV-551               ACCATGTGATCTGCCATCAACCGAACCTGCGCATCCT   37
ENV-550               ACCATGTGATCTGCCATCAACCGAACCTGCGCATCCT   37
HUM-242               ACCATGTGATCTGCCATCAACCGAACCTGCGCATCCT   37
CF_PA39               ACCATGTGATCTGCCATCAACCGAACCTGCGCATCCT   37
E-19-12686-6-1        ACCATGTGATCTGCCATCAACCGAACCTGCGCATCCT   37
C-18-47465-1-1        ACCATGTGATCTGCCATCAACCGAACCTGCGCATCCT   37
E-19-39885-1-1        ACCATGTGATCTGCCATCAACCGAACCTGCGCATCCT   37
E-19-52481-1-1        ACCATGTGATCTGCCATCAACCGAACCTGCGCATCCT   37
LiA133_2003           ACCATGTGATCTGCCATCAACCGAACCTGCGCATCCT   37
J80UH1OS1             ACCATGTGATCTGCCATCAACCGAACCTGCGCATCCT   37
FADDI-PA107           ACCATGTGATCTGCCATCAACCGAACCTGCGCATCCT   37
WH-SGI-V-07050        ACCATGTGATCTGCCATCAACCGAACCTGCGCATCCT   37
17896/7/2             ACCATGTGATCTGCCATCAACCGAACCTGCGCATCCT   37
178967/1              ACCATGTGATCTGCCATCAACCGAACCTGCGCATCCT   37
                      **** ** ******** ** *****************
```

Table S1.- Bacterial strains, plasmids and oligonucleotides used in this study		
**Strain**	**Description**	**Reference**
E. coli DH5α	Nal^{R}; *F- φ80lacZΔM15, Δ(lacZYA-argF) U169, recA1, endA1, hsdR17 (rk^{-}, mk^{+}), phoA, supE44, λ^{-}, thi-1, gyrA96, relA1.*	Invitrogen ®
E. coli S17-1 λpir	Sm^{R}; *thi pro hsdR^{-} hsdM^{+} recA SmR [RP4-2 (Tc::Mu) (Km::Tn7)] λpir*	Qiagen ®
E. coli XL1-Blue	Tc^{R}; *endA1 gyrA96(nalR) thi-1 recA1 relA1 lac glnV44 F'[::Tn10 proAB^{+} lacIq Δ(lacZ)M15] hsdR17(rK^{-} mK^{+})*	Stratagene ®
Pseudomonas aeruginosa		
PAO1	Wild type strain of the MPAO1 sub-line isolated in Melbourne, Australia from an infected wound.	(Holloway B. W., 1955)
PAO*Δvfr*	Apra^{R}; PAO1-derived Δ*vfr* mutant strain.	This work
Pseudomonas paraeruginosa		
MAZ105	Wild type strain isolated from tomato rhizosphere at Guerrero, México.	(Quiroz-Morales et al., 2023)
**Plasmid**	**Description**	**Reference**
pLP170	Cb^{R}; a vector designed for the construction of transcriptional fusions, incorporating the *lacZ* reporter gene.	(Preston et al., 1997)
pLPpqsE^{PAO1}	Cb^{R}; a 352 bp DNA fragment encompassing the promoter region of the *pqsE* gene from the PAO1 strain, cloned into the EcoRI and HindIII restriction sites of the pLP170 vector.	This work
pLPpqsE^{MAZ105}	Cb^{R}; a 352 bp DNA fragment encompassing the promoter region of the *pqsE* gene from the MAZ105 strain, cloned into the EcoRI and HindIII restriction sites of the pLP170 vector.	This work
pLPEMut	Cb^{R}; pLPpqsE^{PAO1} derived plasmid containing point mutations in the Vfr-binding site (T246C and G243C) relative to the ATG translational start site of *pqsE* gene.	This work
pIJ773	aac(3)IV(Apra^{R}), *oriT*$^{+}$ plasmid. Source of the apramycin resistant cassette	(Gust et al., 2003)
pJET2.1/blunt	Cb^{R}; cloning vector that carries a lethal restriction enzyme gene that it is disrupted by ligation of a DNA insert into the multicloning site.	Thermo-Fisher ®
pJETvfr::Apra	Cb^{R}; Apra^{R}; pJET2.1/blunt-derived plasmid encompassing the deletion allele to replace the structural *vfr* gene with the apramycin resistance cassette by double homologous recombination.	This work
pBBR1MCS	Tc^{R} broad host range cloning vector containing a multiple containing a multiple cloning within the lacZα gene	(Kovach et al., 1995)
pBBR1MCS-vfr	TcR PBBR1MCS derived plasmid carrying the vfr gene under the control of the lac promotor.	This work

**Construction**	**Oligonucleotide name**	**Sequence 5'→3'**
pLPpqsE^{PAO1}	1233-25_5TsE1pqsEFw<br>1234-25_3TsH3pqsERv	AGCGAATTCCCGACCTTCCTCGACGA<br>CGTAAGCTTTCGGAGCCGTAGGTCAGG
pLPpqsE^{PAO1}	1233-25_5TsE1pqsEFw<br>1234-25_3TsH3pqsERv	AGCGAATTCCCGACCTTCCTCGACGA<br>CGTAAGCTTTCGGAGCCGTAGGTCAGG
pLPpqsE^{MAZ105}	1233-25_5TsE1pqsEFw<br>1234-25_3TsH3pqsERv	AGCGAATTCCCGACCTTCCTCGACGA<br>CGTAAGCTTTCGGAGCCGTAGGTCAGG
pLPEMut	3095-25_mut1FwpLPpqsE<br>3096-25_mut1RvpLPpqsE	GACGACATCGACCACGTCATCTGCCATCAACC<br>GGTTGATGGCAGATGACGTGGTCGATGTCGTC
Apramicine resistance cassette	9522-14_ApraFw<br>9523-14_ApraRv	TATTCCGGGGATCCGTCGAC<br>TGTAGGCTGGAGCTGCTTC
	1879-25_H3UpvfrFw	CCAAGCTTGAGCAGTATTTCTCCGCCGA

pJETvfr::Apra	1880-25_vfr5ApraRv	gtcgacggatccccggaataATAGCTACCATGCCCGAGTC
	1881-25_ vfr3ApraFw<br>1882-25_H3DwvfrRv	gaagcagctccagcctacaTTTTCATGGGGTTCGACAGC<br>CCAAGCTTCTATCTGAAGGAAGCGCGGG
pBBR1MCS-vfr	3377_26_Fw_PlacvfrK<br>3378_26_Rv_vfr_X	ATCGGTACCtttacactttatgcttccggctcgtatgttgAGGTCTCTCCATAAGAACTC<br>.ACTTCTAGACCTTTTTCATGGGTGCTG
**Antibiotics resistance abbreviations: Nal^{R} (nalidixic acid), Sm^{R} (streptomycin), Tc^{R} (tetracycline), Apra^{R} (apramycin), Cb^{R} (carbenicillin)**		

References

Gust B, Challis GL, Fowler K, Kieser T, Chater KF. PCR-targeted *Streptomyces* gene replacement identifies a protein domain needed for biosynthesis of the sesquiterpene soil odor geosmin. Proc Natl Acad Sci U S A. 2003;100(4):1541–1546. doi:10.1073/pnas.0337542100.

Holloway BW. Genetic recombination in Pseudomonas aeruginosa. J Gen Microbiol. 1955;13(3):572–581. doi:10.1099/00221287-13-3-572.

Kovach ME, Elzer PH, Hill DS, Robertson GT, Farris MA, Roop RM 2nd, Peterson KM. Four new derivatives of the broad-host-range cloning vector pBBR1MCS, carrying different antibiotic-resistance cassettes. Gene. 1995;166(1):175–176. doi: 10.1016/0378-1119(95)00584-1.

Preston MJ, Seed PC, Toder DS, Iglewski BH, Ohman DE, Gustin JK, Goldberg JB, Pier GB. Contribution of proteases and LasR to the virulence of *Pseudomonas aeruginosa* during corneal infections. Infection and immunity. 1997;65(8):3086–3090. doi: 10.1128/iai.65.8.3086-3090.

Quiroz-Morales SE, Muriel-Millán LF, Ponce-Soto GY, González-Valdez A, Castillo-Juárez I, Servín-González L, Soberón-Chávez G. *Pseudomonas aeruginosa* strains belonging to phylogroup 3 frequently exhibit an atypical quorum sensing response: the case of MAZ105, a tomato rhizosphere isolate. Microbiol (Reading). 2023;169(10):001401. doi: 10.1099/mic.0.001401.
